# Unlocking community capability through promotion of self-help for health: experience from Chakaria, Bangladesh

**DOI:** 10.1186/s12913-016-1865-9

**Published:** 2016-11-15

**Authors:** Abbas Bhuiya, Syed Manzoor Ahmed Hanifi, Shahidul Hoque

**Affiliations:** 1 Partners in Population and Development, Dhaka, Bangladesh; 2 Health Systems and Population Studies Division, International Centre for Diarrhoeal Disease Research, Bangladesh (icddr,b), Dhaka, 1212 Bangladesh; 3 Formerly of Health Systems and Population Studies Division, International Centre for Diarrhoeal Disease Research, Bangladesh (icddr,b), Dhaka, 1212 Bangladesh

**Keywords:** Self-help, Community participation, Community capability, Participatory research, Chakaria, Bangladesh

## Abstract

**Background:**

People’s participation in health, enshrined in the 1978 Alma Ata declaration, seeks to tap into community capability for better health and empowerment. One mechanism to promote participation in health is through participatory action research (PAR) methods. Beginning in 1994, the Bangladeshi research organization ICDDR,B implemented a project “self-help for health,” to work with existing rural self-help organizations (SHOs). SHOs are organizations formed by villagers for their well-being through their own initiatives without external material help. This paper describes the project’s implementation, impact, and reflective learnings.

**Methods:**

Following a self-help conceptual framework and PAR, the project focused on building the capacity of SHOs and their members through training on organizational issues, imparting health literacy, and supporting participatory planning and monitoring. Quarterly activity reports and process documentation were the main sources of qualitative data used for this paper, enabling documentation of changes in organizational issues, as well as the number and nature of initiatives taken by the SHOs in the intervention area. Health and demographic surveillance system (HDSS) data from intervention and comparison areas since 1999 allowed assessment of changes in health indicators over time.

**Results:**

Villagers and members of the SHOs actively participated in the self-help activities. SHO functionality increased in the intervention area, in terms of improved organizational processes and planned health activities. These included most notably in convening more regular meetings, identifying community needs, developing and implementing action plans, and monitoring progress and impact. Between 1999 and 2015, while decreases in infant mortality and increases in utilization of at least one antenatal care visit occurred similarly in intervention and comparison areas, increases in immunization, skilled birth attendance, facility deliveries and sanitary latrines were substantially more in intervention than comparison areas.

**Conclusion:**

Building community capability by working with pre-existing SHOs, encouraging them to place health on their agendas, strengthening their functioning and implementation of health activities led to sustained improvements in utilization of services for over 20 years. Key elements underpinning success include efforts to build and maintain trust, ensuring social inclusion in project activities, and balancing demands for material resources with flexibility to be responsive to community needs.

## Background

Individuals and groups interact, formally and informally, within health systems to ideally improve the health of those living in communities. These interactions facilitated by active community engagement or participation can enhance the utilization of health services [1, 2]. For example, the success of the Expanded Program on Immunization (EPI) programme in Bangladesh in increasing immunization coverage from 6 % in 1986 to over 75 % in 1991 was only possible with active community participation [3, 4]. Other examples abound detailing the effects of community participation for improved development outcomes including health, water, sanitation and agriculture [5, 6].

One way to facilitate community participation is by using Participatory Rural Appraisal (PRA) [7–9]. The continuum of PRA varies from engaging community members solely in data collection to supporting community use of data collected to understand a phenomenon, such as health seeking behaviour, and design programmes for solving the challenges faced [10]. In most cases of participatory research, community members are considered local experts and researchers attempt to learn from them. In the process, community members may also learn something from the researchers either as groups or individuals. Ideally these exchanges between community members and researchers as outsiders build community capability, but this is not always the case [5, 11].

Despite the recognition given to community participation and to PRA for sustained health and development, documentation of such initiatives over the long term and their impact on unlocking community capability is not common in the literature [12]. It is against this background that this paper attempts to document experience from ongoing program efforts to support community participation following PRA starting from the mid-90s in a rural area of Bangladesh. We first describe the local context of Chakaria in rural Bangladesh and the ways in which efforts were designed, implemented and monitored to facilitate community participation and to empower communities to address their own problems. We present outcomes in terms of changes in the organizational practices of self-help organizations (SHOs), as well as the impact of strengthening community capability on health in Chakaria [13].

## Methods

### Study area

Chakaria is located in the southeast coast of the Bay of Bengal. The location of Chakaria has made it very vulnerable to cyclones and tidal waves in addition to regular monsoon flooding. Climate change has heightened the risk of extreme weather events. Administratively, Chakaria is an *Upazila* (sub-district) with a population of about 474,000 (as of 2011) spread across terrain of which one fifth consists of rivers and canals [14–16]. The main economic activities in the area are agriculture, forestry, and sea-fishing, with shrimp farming recently developed mainly for the international market. Thirty percent of households are landless and about half depend on income from menial labour. Ninety-one percent of the population in Chakaria are Muslim, with the remainder being Hindu or Buddhist. The study site is one of the most religiously conservative areas in Bangladesh, with very low levels of secular education. The literacy rate among population aged 7 years or more is 47.6 % with similar levels for men and women.

In 1994, at the time of starting our efforts, Chakaria *Upazila* had one 31 bed government hospital located at the *Upazila* headquarters, two Health and Family Welfare Centres at the union level and two Rural Dispensaries [17]. In 2014, the scenario was quite different: the government hospital had upgraded to 50 beds, there were 11 Health and Family Welfare Centres, and 23 Community Clinics [18], as well as two private clinics.

### Self-help approach for unlocking community capability

**Fig. 1:**
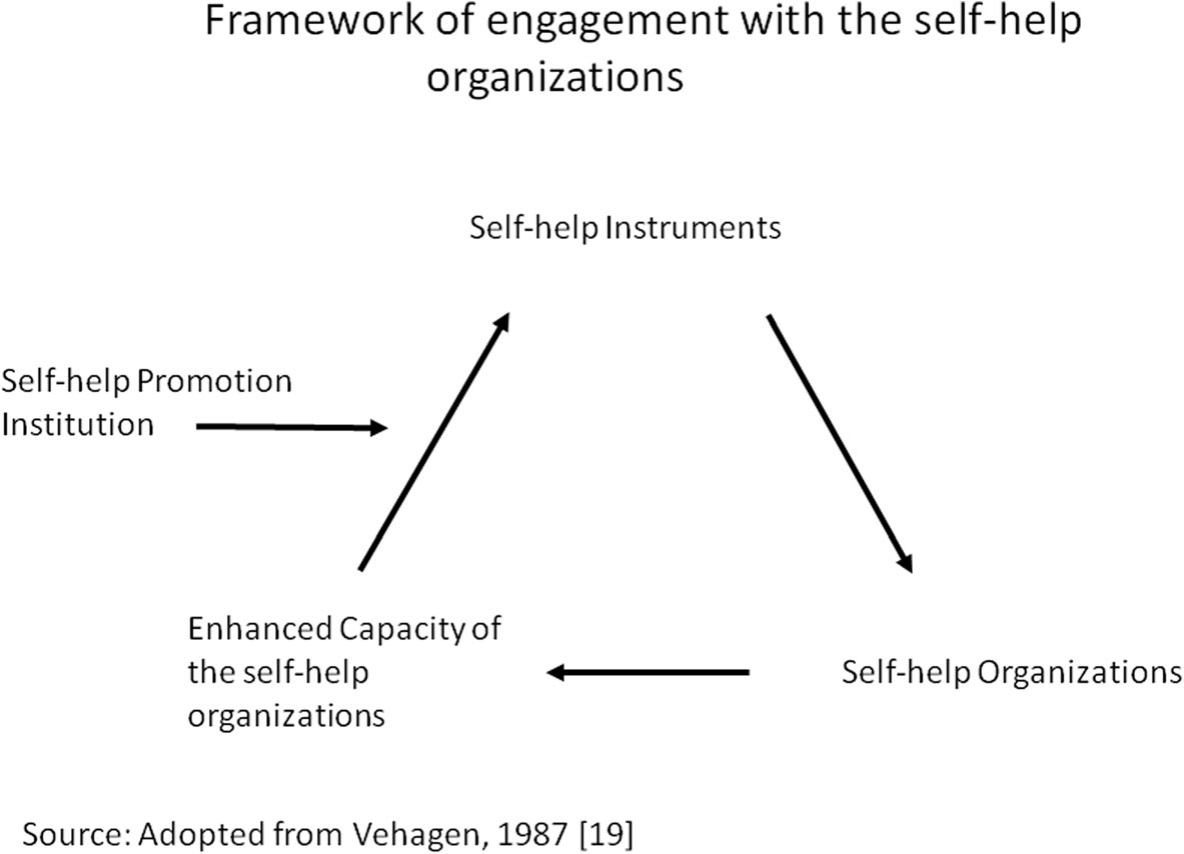
Framework of engagement with the self-help organizations

The focus of ICDDR,B’s efforts to strengthen community capability from 1994 until 2006 through promotion of self-help for health at both individual and collective levels was based on the framework (Fig. 1) proposed by Verhagen and Cebemo [19].

At the individual level, the focus was on raising awareness related to health promotion, prevention and health care seeking. At the collective level, ICDDR,B worked with existing SHOs to enhance their capacity to tackle community health issues. SHOs are organizations formed by villagers for their well-being through their own initiative without external help. Examples of SHOs include village clubs, youth clubs, profession based associations and cooperatives, and mosque committees. ICDDR,B support to SHOs involved three phases: (1) getting to know the community and building relationships; (2) conducting a needs assessment and designing activities to address the needs identified, and (3) implementation. A summary of the range of PRA techniques used and their purposes are presented in Table 1. Throughout, care was taken to not create a dependency relationship with community members. Hence no material resources were given and no new organizational structures were created. Villagers were involved in these activities right from the start to enhance and build a relationship of mutual trust and to lay the foundation for future processes of community mobilization where they would lead initiatives [20].

**Table 1:** List of PRA techniques used

PRA tools used	Purpose
Transect	Knowing the area, building relationships, and spreading the word about the project.
Social Map	Understanding the social structure, assessing the available institutional resources e.g., SHOs, health facilities.
Seasonal Calendar	Seasonal dimension of livelihood, workload, farming, food availability, human diseases, gender-specific income and expenditure.
Daily activity clock	Knowing the pattern of villagers’ daily activities. Particularly, for looking at relative workloads of different groups in the community. Identifying suitable time for the availability of the villagers.
Venn Diagram	Portraying the importance of various health problems and utilization of health services.
Village history	Bringing a historical perspective of change and development, and that change is always a part of societal development.
Focus Group Discussion	Identifying major health problems, ranking the problems in terms of perceived importance, and identifying perceived causes, and discussing scientific causes and possible solutions.
People’s Participatory Planning	Developing a work plan with a time line, assigning who will do what, identifying progress monitoring indicators.
Participatory Impact Monitoring and Evaluation	Monitoring impact of SHO activities, as a routine work, using data collected by the SHO members, and analysed with the help of the project staff members.

The functioning of village health posts, which was an outcome of community initiatives triggered by the ICDDR,B programme, continued by the SHOs for five years from 1999 to 2004 and then had some slackness until 2010 due to reduced support from ICDDR,B because of lack of funding. ICDDR,B inputs to promote self-help were stopped after 2010 other than implementing a micro-health insurance in the area with village health posts being the point of care.

The ICDDR,B project team, overseen by one project director, was comprised of two groups that worked independently from each other: implementers and those conducting monitoring and evaluation. Some of those involved in monitoring and evaluation were also involved in the Health and Demographic Surveillance System (HDSS). The project team members involved in implementation were trained extensively in PRA methods both at a theoretical level in classes and also through practical experience in communities.

### Knowing the community and building confident relationships

#### Transect analysis

To understand the community and to establish a confident relation with the community, ICDDR,B staff members started with a transect analysis. This involved walking through the villages, getting introduced to people and explaining the purposes of the project in a very informal way. The entry point was talking in common meeting places, such as shops, about common health problems faced by the villagers and ways the villagers manage them. Information about the number of schools, mosques, clubs, other community organizations, and local key and resource persons were also gathered during these informal visits.

#### Participating in social events

Participation in social events as they occur in communities facilitated outside entry into local social systems, enabling familiarity with community members, and building relationships with trust with them. ICDDR,B staff members participated in social events like, *Janaja* (funeral prayer for deceased person), sports and cultural functions, *Milad* (religious gatherings), *Juma* (Friday) prayers and other important social events.

#### Participating in government programmes

Project staff supported community health by participating in government programmes such as EPI sessions and national immunization days. Staff members also participated, on invitation from high schools (6th to 10th grade), in school maternal and child health sessions held in observance of maternal and child health weeks declared by the government. Subsequently, school children spoke highly of the project staff and the sessions to their parents, thereby facilitating further trust building and relationships between project staff and families [20]. This experience also helped project staff realize the importance of school based health education, which was later taken up by ICDDR,B.

#### Identification of SHOs and key and resource persons

Traditionally Bangladeshi society has been rich in community organization and initiatives; Chakaria was not an exception to this. A list of self-help organizations along with their contact persons was prepared based on the transect walks, individual and group discussions, and subsequent visits made to the organizations and local key and resource persons. Organizations were selected that had highly representative membership, broad community support, regular meetings, ongoing activities, and resources at their disposal. Attempts were made to keep in touch with organizations and key resource persons that were not selected for active engagement to avoid misunderstanding.

### Meetings with SHOs and bringing health onto their agenda

Project staff started to participate in the routine meetings of selected SHOs and observed the procedures followed in their meetings. None of the organizations initially had health on their agenda. Project staff requested permission from SHO leaders to discuss the major problems people faced. In most cases SHO members identified economic, social and religious well-being as their priority. After intensive discussion between project staff and SHO members, SHO members concluded that good health was also a priority as it was needed for everything, including prayers, education and any activity for making a living.

Discussions with SHO members indicated that although villagers took initiative to address local issues, they did not perceive a role for themselves in health. Preventive measures, such as immunization and epidemic control, was viewed solely as a government responsibility, while curative care was an individuals’ responsibility, managed through consulting with healthcare providers and therefore not perceived as a collective concern [20]. The issue of preventable diseases was raised and discussed, such as the issue of transmission of diarrhea. Villagers were not aware of the causes and routes of transmission of diarrhea. The advantages of preventive health behaviour, and the adverse consequences of sickness on health and economic well-being was further emphasized during subsequent interactions.

Meetings were concluded with the inclusion of health in the SHOs’ agendas and an expression of willingness by the organizations to get engaged in health related activities. It was decided that SHO members would go back and discuss the meeting outcomes with other SHO members who were not present in the meeting. If they considered that the issues discussed should be addressed, they needed to find ways to incorporate them into their activities. ICDDR,B was willing to provide technical and planning support in this regard, if the organizations desired.

### Participatory needs assessment and monitoring

#### Knowing health seeking behaviour

Wealth ranking and mobility mapping were used to understand people’s health seeking behaviour and its relationship with household socioeconomic status. SHO members conducted wealth ranking by classifying households into high, medium and low categories in terms of socioeconomic status. They also drew maps that helped to understand the location of households and nearby health facilities and what kind of health services people resorted to when people became sick in both dry and monsoon seasons. These exercises helped to understand how the utilization of services was linked with household wealth ranking and whether existing health services were accessible or adequate.

#### People’s participatory planning

While SHOs had the experience of making plans, they were new to participatory planning and required orientation. Project staff first familiarised SHO representatives with the common health issues and problems prevailing in their communities to ensure that participatory planning sessions focussed on health and health related issues. Subsequently SHOs convened meetings to carry out participatory planning and invited project staff to help facilitate the first couple of sessions.

Participatory planning started with recollecting the past health and development challenges people faced. In most cases the challenges they faced were socioeconomic and developmental in nature. Examples of health challenges included epidemics of cholera, smallpox and outbreak of malaria and other prevalent disease conditions, environmental health issues and harmful practices. This was followed by participatory discussion of root causes of the problems they faced earlier. At the third stage the participants discussed the possible ways to tackle the problems and what resources they had for this purpose. In their opinion communities had human resources (government health workers and village doctors), who could be trained to enhance knowledge and capability on health and development issues, and carry out the health education activities. At the final stage of participatory planning they focused on the future and made short and long term action plans.

#### Participation of women and the poor

Analysis of the monitoring data by the project team showed that in most of the participatory planning sessions, only male members participated, and although male villagers from poorer families participated, their voices were not heard. The issue was raised by project facilitators at the participatory planning meetings while presenting data on who participated and who did not. After discussion, SHO members felt the need to support participation by women and poor members in health development activities. They also noted the problem of social exclusion in the school health education programme which did not include children from very poor households because children from poorer families did not attend schools [21]. In contrast in their opinion, mosque-based programs while exclusive to males, did include members from the poor households.

Due to religious and social sentiments, which limited the mobility of women and sustained a highly hierarchical society, it was agreed that women and poorer community members should be included in health activities through a separate process. Health education for women in clusters of households was started with female volunteers. In addition, women’s groups formed by development non-governmental organizations (NGOs) for the poorest of the poor were also linked to the SHOs to work collaboratively.

### Implementation

#### Training of SHO volunteers and dissemination of health messages

Considering the importance of health knowledge, SHOs decided through participatory planning to train SHO volunteers. Nearly 4,500 volunteers selected by the SHOs were trained and subsequently conducted health awareness sessions throughout the year. Three types of volunteers were identified for training: Male Health Volunteers (831), Female Health Volunteers (1743), School Health Volunteers (1698 both male and female), and Village Health Post Volunteers (177), both male and female. The trained volunteers disseminated health message to respective target populations as indicated below.

- Male Health Volunteers: Disseminated health educational messages to men in male-dominated spaces, such as mosques, tea stalls, clubs, group meetings, village gatherings, gatherings in the neighbourhoods. This health message dissemination was done in an informal manner following a guideline developed by the SHOs with technical support from project staff members.
- Female Health Volunteers: Disseminated health messages using flip charts among women in their neighbourhoods when the women assembled in leisure time.
- School Health Volunteers: Students of grade six to 10 disseminated health messages among classmates in schools, playmates in the neighbourhood, family members and other peer group members they found appropriate. This involved role playing wherever appropriate.
- Village Health Post Volunteers: Adult male and female villagers nominated by the SHOs to help manage the operation of the village health posts. This group was formed after the establishment of the village health posts.

After 2 years of health promotion activities the SHO representatives raised the issue that despite all the health education, people still got sick and pregnant women continued to suffer as local health care providers lacked training. SHO members wanted to establish primary care centres in the villages, and also wanted their women trained in community midwifery. While the project did not have any mission to get involved in clinical services, ICDDR,B staff agreed to provide technical assistance by deploying six paramedics, a government recognized cadre of health professionals having a formal diploma in healthcare, for six village posts established by SHOs. The health posts were built with resources mobilized by the SHOs on land donated by a villager. SHOs also identified 15 women from their villages for a 6-month residential course on midwifery with project resources.

### Participatory impact monitoring and evaluation

SHOs established a system to monitor progress and share information between partners, i.e. from project to SHO representatives and also from SHOs to project staff. SHO representatives were oriented to conduct Participatory Impact Monitoring and Evaluation. Volunteers collected information on EPI, sanitary latrines, and other related information decided in consultation with the project team. SHO members compiled the data, initially with help from project staff, and presented it in SHO meetings, where SHO members and villagers participated and analysed the information. This became a regular activity that encouraged SHOs to meet the targets set in their work plans and created an environment of data based decision making.

### Study design and study period

The self-help for health program was implemented by following a quasi-experimental study design with an intervention area (population 125,796 in 1999) and a comparison area (population 40,409 in 1999). Intervention and comparison areas were selected on the basis of similarity of socioeconomic background and from the neighbouring area within the sub district to ensure comparability as much as possible [22]. The comparison area had all the available government and other services available and the project did not interfere with any new services being made available in the comparison area.

### Data sources

Data used in this paper came from surveys carried out in various points in time, project process documents, work plans, project activity reports, and published and unpublished materials. Data for health and demographic outcomes came from the HDSS, which has been in operation since 1999. HDSS, in 1999, included 20,252 and 6,727 randomly chosen households in the intervention and comparison areas respectively [22]. The project implementation design allowed an assessment of long-term effects of self-help approach on community initiatives and responses post 2003 up to 2015. All findings were first shared with the representatives of the SHOs in their monthly meetings.

## Results

### Change in the organizational management practices

Based on the organizational capacity building activities offered by the project, SHOs were able to strengthen their internal organizational management and also how they planned and undertook activities (Tables 2 and 3). Over time, the SHO leadership not only appreciated the value of formalising and systematizing their organizational functioning, but these processes also earned them further confidence from community members.

**Table 2:** SHO functionality overtime based on project reports

Before project efforts	After project efforts
No specified action plan as an outcome of the meetings	Yearly action plans with time bound monitoring indicators
No systematic monitoring and evaluation system	Monitoring system in place
Issue based fund collection and informal way of holding and managing funds	Regular fund collection and opening bank accounts to deposit collections and formal process of decision for spending money and record keeping
No plan for leadership and organizational development	Plan for organizational development & leadership was part of work plan

**Table 3:** Characteristics of SHOs overtime as measured by project surveys

Characteristics	1994	2015
Total number of SHOs in intervention area	45	93
Holding executive committee	95 %	100 %
Convened meeting regularly	36 %	65 %
Had written by-laws	78 %	67 %
Written meeting minutes regularly	71 %	65 %
Prepared annual financial report	70 %	82 %
Health matters in agenda in the regular meeting	0 %	42 %

As detailed in Table 3, the numbers of SHOs doubled between 1994 and 2015 with 51 new SHOs formed. We are not able to determine whether this increase was due to the project or due to a secular trend. Among the 45 organizations listed in 1994, 11 stopped their activities due to various reasons, which included migration of a critical executive committee member and/or merger with other organizations. While most of the organizational characteristics show improvement over time, the proportion of SHOs having written by-laws and regular meeting minutes decreased slightly, although the absolute numbers increased over time. Of note, none of the SHOs had health on their agenda in 1994, but in 2015, 42 % of SHOs reported that their agenda included health.

### Health initiatives by the SHOs and community members

**Fig. 2:**
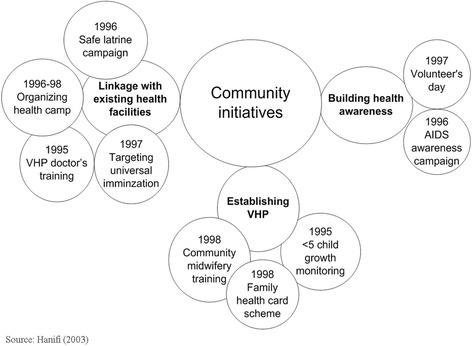
Some of the health activities undertaken by SHOs

Some of the major health initiatives undertaken by SHOs and community members during 1994–2003 and post 2003 are detailed in Table 4 and in Fig. 2 (adapted from Hanifi [23]).

**Table 4:** List of initiatives taken by SHOs and their outcomes, 1994–2015, based on project documentation

Initiatives taken by the SHOs	Actions	Outcome
1994–2003		
Diarrhoea Epidemic Control	Pushed the local authority to control the epidemic	Epidemic was controlled; permanent oral rehydration treatment depot established; SHOs gained confidence in collective action
Campaign for Promotion of Sanitary Latrines	House to house campaign to setup sanitary latrines	Increase ownership of sanitary latrines; reduced incidents of related disease; market based latrine production unit established
Campaign to promote hand washing	SHO members campaigned for proper hand washing after defecation and before meals	90 % of households found using ashes or soap; diarrhoea and other related disease reduced; continued healthy practices.
Campaign to promote practice of covering food	Traders at the markets were pushed not to sell unhygienic foods and to cover food after preparation	Foods were seen covered at shops; reduced selling of unsafe food items; established social responsibility
Union Health Committee	Formed a coordination body comprising representatives from all SHOs in a union	Coordination of all health and development activities at union level
Village Health Posts [24]	SHOs and Union committees established 7 Village Health Posts to supplement public health services with their own resources	Village Health Posts were sustained for long time (1998–2016); became a community focal point for health services and social activities.
Family health card scheme	A low cost family health card scheme initiated	People were getting benefit from the scheme; 6 % of households enrolled; The scheme later was developed into micro-health insurance.
Fund for poor people	SHO members felt need to have fund to help the poor, they raise a fund through contribution from people interested to donate	Poor people getting help from the fund for health and other needs
Health facility monitoring and utilization	SHOs established a system to monitor government and non-government health facilities	The facilities were monitored regularly; utilization of the facilities ensured; linkage with Government of Bangladesh authorities and committees established
Health camps	Health camps were regularly organized by SHOs/Village Health Post committees for treatment	Ear Nose and Throat, Circumcisions and other special camps were organized regularly
Traditional Birth Attendants’ (TBAs) Training	Training for TBAs were organized by the SHOs and supervision provided in the form of refresher training and advice from project physicians	More than 50 TBAs trained by a hospital near Dhaka, the capital of Bangladesh. Fee and travel costs were provided by the project.
Bed net program [25]	With government help the bed net program was initiated	Malaria incident decreased; program run by national NGOs based on SHO experience
Training village doctors	Committees felt the need to train village doctors. SHOs arranged financial assistance to train village doctors from *Upazila* Health Complex	Village doctors were actively giving services; regular training uptakes by ICDDR,B as part of other program.
Training of women as skilled birth attendant (SBA) [26]	Community demand skilled personnel for assisting deliveries	14 were trained as community midwives; they are still giving services the community
AIDS Awareness Campaign [27]	SHO volunteers carried out the campaign	Increase of knowledge reported and disseminated as per ICDDR,B records
2004–2015		
Running the village health posts in the absence external financial support	SHOs kept the village health posts established earlier running by allowing the community midwives and trained village doctors to provide services from them.	Village health posts continued to be the place of lowest primary healthcare for the villagers.
Promoting and supporting the community midwives trained during the earlier phase	Community midwives got a room at the village health posts to render their services regularly	Pregnant women are getting benefits from the midwife services
Promoting and supporting the trained village doctors [28]	Trained village doctors were involved in services of village health posts	Trained village doctors promoting telemedicine services
Supporting and promoting a voluntary micro-health insurance programme	Micro-health insurance services were running from the village health posts and the committees is in steering role	People enrolling into the scheme

### Health outcomes and healthcare utilization

The self-help for health project aimed to improve the health status of the villagers by increasing collective health promotional actions and utilization of modern healthcare services. Changes in some of the relevant indicators in the intervention and comparison areas are presented below.

### Infant mortality

**Fig. 3:**
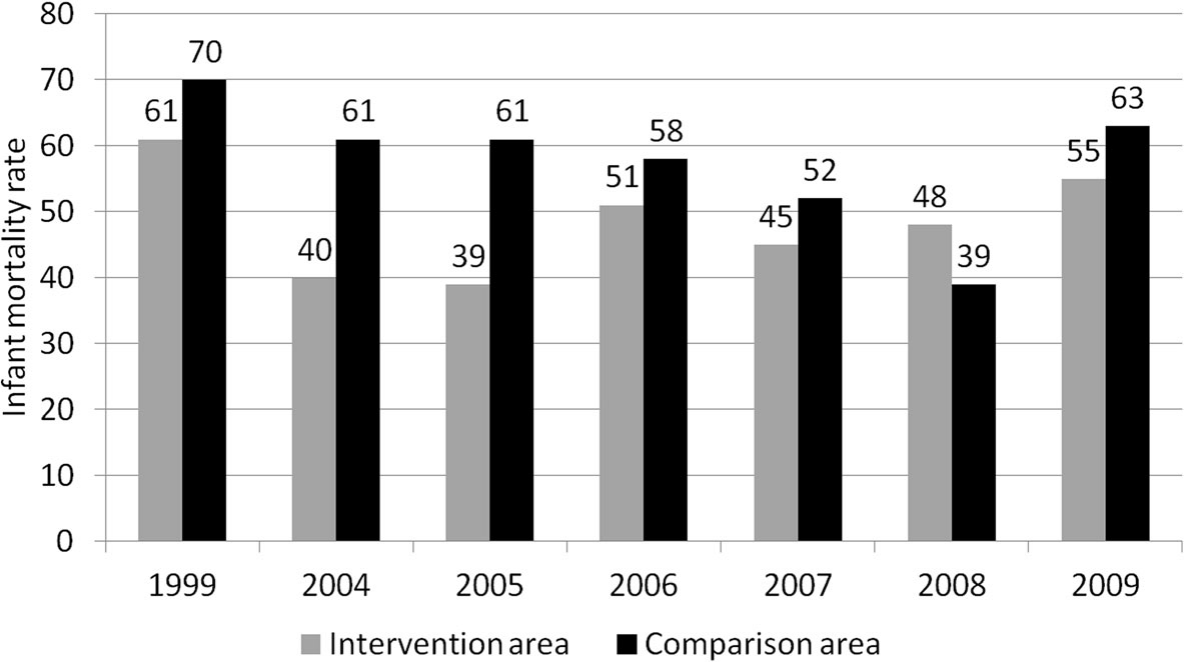
Infant mortality rate by area

While infant mortality data were not available for the period 2000 to 2003, a larger decline in infant mortality rate was seen between 1999 and 2000 in intervention areas vs. comparison areas, during the most active period of the project. Subsequently, these gains were lost [29, 30] and overall there was no substantial difference in declines in infant mortality between 1999 and 2009 between intervention and comparison areas (Fig. 3).

### Immunization

**Fig. 4:**
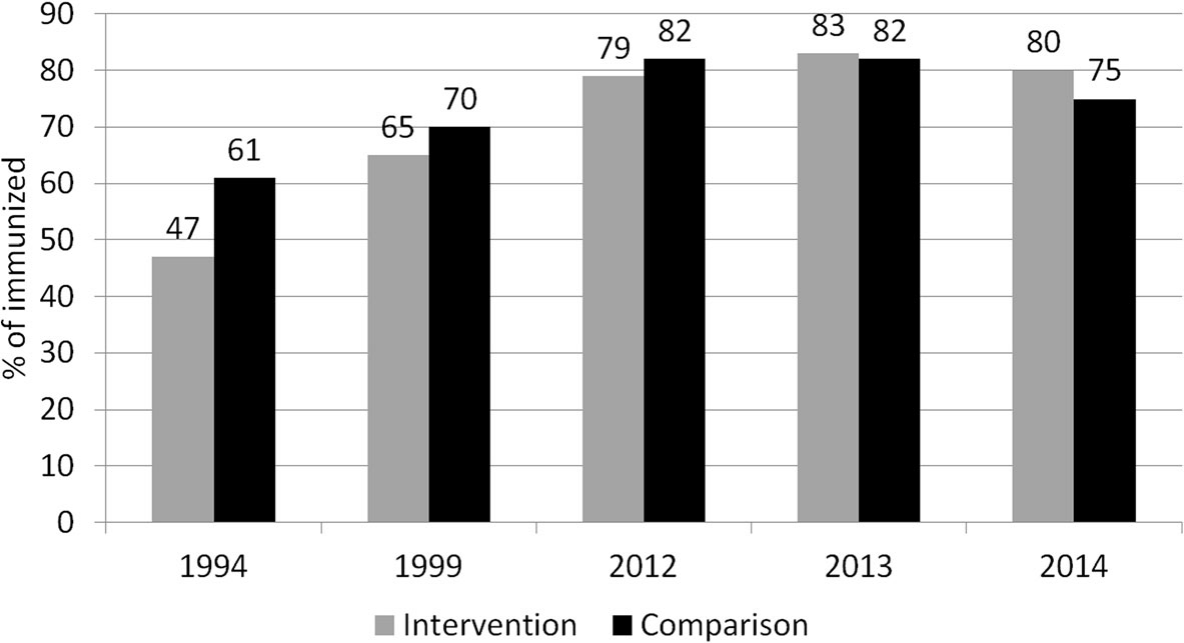
Percentage of children received five routine vaccines (BCG, DTP1-3, Measles vaccine)

The percentage of fully immunized children in the intervention area increased substantially more from 47 % in 1994 to 80 % in 2014, compared to 61 % in 1994 to 75 % in 2014 in the comparison area (Fig. 4). The self-help program resulted in collective action supporting more regularly held and better utilized immunization services, by addressing negative attitudes towards immunization and mobilizing people to inform villagers about immunization sessions.

### Safe delivery

**Fig. 5:**
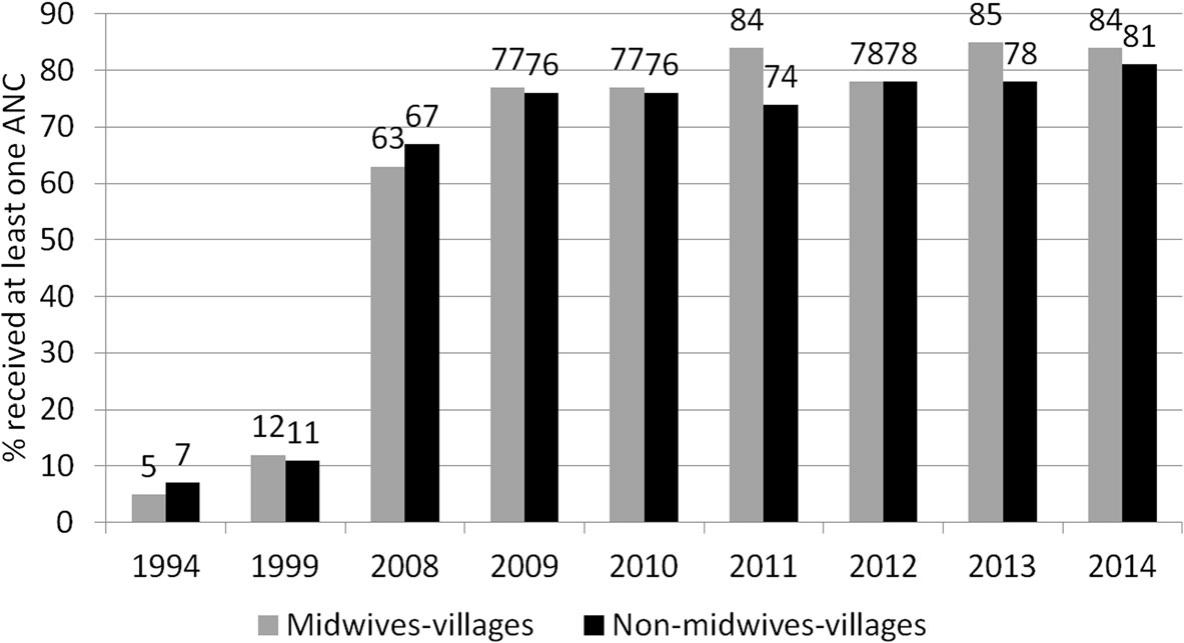
Percentage of women received at least one ANC

**Fig. 6:**
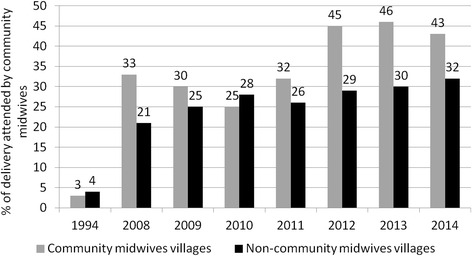
Percentage of deliveries assisted by community midwives

**Fig. 7:**
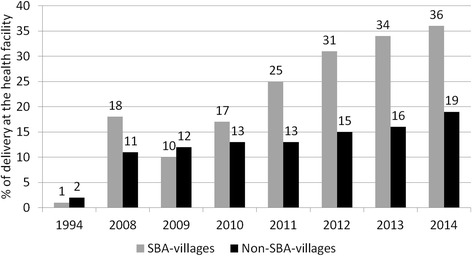
Percentage of deliveries took place at health facility

Utilization of at least one ANC services increased in both intervention and comparison areas substantially from 5 and 7 % in 1994 to 84 and 81 % in 2014 (Fig. 5). In contrast, during the same time period while use of community midwives increased delivery by a skilled birth attendant (SBA) and facility deliveries more in intervention areas (3 to 43 % SBA, 1 to 36 % facility deliveries) than in comparison areas (4 to 32 % SBA, 2 to 19 % facility deliveries), substantial work remains to improve coverage in both areas (Figs. 6 and 7).

### Child nutrition

An important focus of work carried out by SHOs was to monitor child nutrition and growth through village health posts and advise caregivers accordingly. As a part of data collection by the project, mid-upper arm circumference (MUAC) of children was collected in the HDSS area in 1994 and 1999 [31, 32]. The proportion of severely malnourished children decreased in both the areas – although the decrease was higher in the intervention area compared to comparison area (Table 5).

**Table 5:** Distribution of MUAC of children aged 6–23 months, Chakaria, 1994 and 1999

MUAC (cm)	Intervention area		Comparison area	
	1994 (%)	1999 (%)	1994 (%)	1999 (%)
<12.5 (severely malnourished)	38.9	22.0	38.5	28.7
12.5–13.4	29.1	38.6	30.3	35.0
13.5 +	32.7	39.4	31.2	36.3
Mean	12.3	13.2	12.8	13.1
Standard deviation	1.2	1.2	1.3	1.2
Total number of children (N)	499	5025	366	1682

Source of data: Chakaria Health and Demographic Surveillance System

### Sanitary latrine

**Fig. 8:**
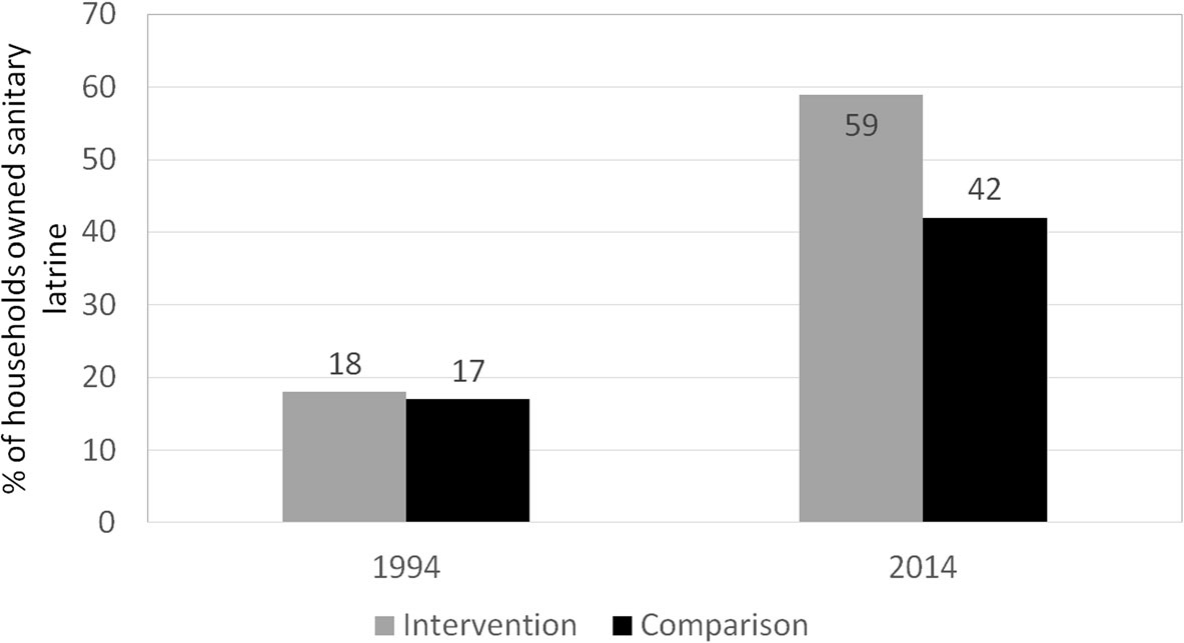
Percentage of household owned sanitary latrine

Information on sanitary latrines and health benefits of the use of sanitary latrines was shared in the meetings of the SHOs and to the villagers through the trained volunteers imparting health messages. This resulted in SHOs taking initiatives to install sanitary latrines in the community and some SHOs also established manufacturing units for construction components of sanitary latrines in the villages. Between 1994 and 2014, this resulted in a higher increase of households with sanitary latrines in the intervention area (18 to 58 %) compared to that in the comparison area (17 to 42 %) (Fig. 8).

## Discussion

The experience gained from supporting health promotion with self-help organizations following Verhagen’s framework [19] and applying PRA has relevance for other similar settings in low-income countries. While initially, the health focus was thought to be challenging, but a substantial proportion of SHOs included health onto their agendas. The proverbial sayings in Bangladesh that “Health is wealth” and “If money is lost, nothing is lost; but if health is lost, everything is lost” underline how health is linked to other livelihood concerns that are central to communities. Despite the successes realized, we also faced challenges in working with communities, which included lack of trust at the beginning, tensions related to including women, demands for material support and for curative services [13, 20]. We conclude this paper by discussing how we faced each of these challenges and reflecting on issues of sustainability.

As mentioned, among the challenges faced, earning community trust was one of the first encountered by project staff. Initially, there was a strong anti-NGO sentiment prevailing in the area, leading project staff to consider deferring its training of volunteers. Instead the project decided to continue with the training plans, but moved the venue from isolated locations to villages where anybody could see and listen what the training was about. In addition to such measures supporting transparency of project actions, building trust required intensive work involving meeting key resource persons at their convenience and explaining the objectives of the project in clear terms, being respectful of community needs as much as possible, and demonstrating a listening mood. Confidence by community members in the project was bolstered by project staff demonstrating reasonableness in decision making and ensuring that what has been said by the project staff was followed through with. Trust was also facilitated by project staff emphasizing community solidarity by including everyone in the village irrespective of social and economic status.

Trust was an outcome of working against social divisions, but also key in facilitating inclusive participation. For example, SHOs initially only nominated men for training. After nearly a year into the participatory planning, the situation was reviewed in SHO meetings on request from the project after having built strong relationships with SHOs. After project staff raising the issue, the SHO members who had exposure to the training programme and who had received feedback from the volunteers realised that the training on health, hygiene, water sanitation, and management of diarrhoea would be more beneficial if women were trained to serve as trainers who could reach other women in the community. This realization led to training of women, which was thought almost impossible at the beginning of the project.

The process of working together not only led to many health activities undertaken by the SHOs, but also the self-realization of community members of their own strength and capability. Moreover, the project played the role of a catalyst to realize the capability of the community for their own wellbeing without creating a dependency relationship between the community and the project. In doing so, the project contested the tendency by villagers of asking for material assistance from outside parties for things which could be managed with their own resources. In some instances, villagers expected project staff to lead activities in their villages -project staff were very particular in communicating that initiatives were not ready to be initiated until villagers were willing to lead them.

Demand for curative services was another challenge because the project was not designed to provide these services. A key learning has been the importance of flexibility in interactions with the SHOs/community members. No blueprint for the project other than the strategy depicted in Fig. 1 was pre-determined excepting the health focus. The project responded by expanding its mandate to support training of village level doctors and midwives, and in other instances by linking SHOs with other relevant agencies for work beyond project mandates.

This paper reflects on the experience of the project in supporting community capability over twenty years. A key feature of our work was our partnership with existing SHOs, instead of forming new groups or approaching community members directly. This helped mitigate the challenge of sustainability after phasing out of the project. As noted through DHSS data, to date some of the health indicators and health care utilization data indicate that positive effects still persist. The strongest of the sustained effects have been the functioning of village health posts, villagers’ response to membership in the recently launched health insurance, and continuation of the project trained community midwives.

In the context of Bangladesh, community clinics, as the lowest level of health facilities, have sought local ownership by way of donating lands. Our experiences highlight that to succeed over the long term, engaging with communities, supporting their capabilities and ownership of community health requires a deeper engagement. Without involving the community in development activities, be it health or other, health planners and managers are unlikely to achieve their expected goals.

## Conclusion

In conclusion, key learnings and recommendations for any initiatives of this kind to unlock community capability has to have the realization that there is no shortcut and that fixed blueprints are unlikely to work. The key is to listen to the community, showing respect to their concerns and jointly acting to tackle them. Wherever possible interventions should be in partnership with existing community organizations for it will not only increase the chances of success, but will also make the change lasting and will strengthen the local level organization of the people. Any investment on community created and owned organizations, empowers and equips the community to tackle future challenges.

## Abbreviations

EPI: Expanded Program on Immunization
HDSS: Health and demographic surveillance system
icddr,b: International Centre for Diarrhoeal Disease Research, Bangladesh
MUAC: Mid-upper arm circumference
NGOs: Non-governmental organizations
PAR: Participatory action research
PRA: Participatory Rural Appraisal
SHOs: Self-help organizations
SBA: Skilled birth attendant
